# Verification of the eighth edition of the UICC‐TNM classification on surgically resected lung adenocarcinoma: Comparison with previous classification in a local center

**DOI:** 10.1002/cnr2.1422

**Published:** 2021-06-24

**Authors:** Hiroshi Minato, Kazuyoshi Katayanagi, Hiroshi Kurumaya, Nobuhiro Tanaka, Hideki Fujimori, Yoshio Tsunezuka, Takeshi Kobayashi

**Affiliations:** ^1^ Department of Diagnostic Pathology Ishikawa Prefectural Central Hospital Kanazawa Ishikawa Japan; ^2^ Department of General Thoracic Surgery Ishikawa Prefectural Central Hospital Kanazawa Ishikawa Japan; ^3^ Department of Diagnostic and Interventional Radiology Ishikawa Prefectural Central Hospital Kanazawa Ishikawa Japan

**Keywords:** adenocarcinoma, lung, subsolid nodules, TNM classification, Union for International Cancer Control (UICC)

## Abstract

**Background:**

The UICC 8th TNM classification of lung cancer has been changed dramatically, especially in measuring methods of T‐desriptors. Different from squamous‐ or small‐cell carcinomas, in which the solid‐ and the invasive‐diameter mostly agree with each other, the diameter of the radiological solid part and that of pathological invasive part in adenocarcinomas often does not match.

**Aim:**

We aimed to determine radiological and pathological tumor diameters of pulmonary adenocarcinomas with clinicopathological factors and evaluate the validity of the 8th edition in comparison with the 7th edition.

**Methods and Results:**

We retrospectively analyzed clinicopathological factors of 429 patients with surgically resected pulmonary adenocarcinomas. The maximum tumor and their solid‐part diameters were measured using thin‐sectioned computed tomography and compared with pathological tumor and invasive diameters. Overall survival (OS) rate was determined using the Kaplan–Meier method for different subgroups of clinicopathological factors. Akaike's information criteria (AIC) was used as a discriminative measure for the univariate Cox model for the 7th and 8th editions. Multivariate Cox regression analysis was performed to explore independent prognostic factors. Correlation coefficients between radiological and pathological diameters in the 7th and 8th editions were 0.911 and 0.888, respectively, without a significant difference. The major reasons for the difference in the 8th edition were the presence of intratumoral fibrosis and papillary growth pattern. The weighted kappa coefficients in the 8th edition were superior those in the 7th edition for both the T and Stage classifications. In the univariate Cox model, AIC levels were the lowest in the 8th edition. Multivariate analysis revealed that age, lymphovascular invasion, pT(8th), and stage were the most important determinants for OS.

**Conclusion:**

The UICC 8th edition is a more discriminative classification than the 7th edition. For subsolid nodules, continuous efforts are necessary to increase the universality of the measurement of solid and invasive diameters.

## 1 INTRODUCTION

Cancer is the world's leading cause of death in humans, and lung cancer has the highest mortality rate among all cancers worldwide. Non‐small cell carcinoma accounts for 85% of lung cancer cases, among which adenocarcinoma is the most prevalent one in recent times.^1^ The strategy for treating lung adenocarcinoma is tremendously important in oncology. In cancer therapy, the tumor‐lymph node‐metastasis (TMN) classification is one of the most important concepts that provides basic information regarding all cancers. The 8th edition of the Union for International Cancer Control (UICC) TNM staging system for lung cancer has been revised extensively, especially in relation to radiological and pathological measurement methods of T factor, which was authorized by the American Joint Cancer Committee (AJCC) on January 1, 2018.^2^, ^3^ The clinical T factor has been changed from the maximum diameter of the tumor to that of the solid part of the tumor, and the pathological T factor from the maximum diameter of the tumor to that of the invasive component. In addition, T factor was further subdivided; T1 and T2 were classified as T1a, T1b, T1c, T2a, and T2b in 1‐cm increments, and T1mi was newly added with the solid part or invasive size being less than 5 mm. Tis was also applied for adenocarcinoma. The new classification applies to all histological subtypes. In cases of squamous cell carcinomas or small cell‐ and large cell‐undifferentiated carcinomas, the diameters of the tumor size, the solid part, and the invasive part mostly agree with each other, such that the determination of T factor is apparently not problematic both radiologically and pathologically. However, adenocarcinoma often contains lepidic components and fibrous scars, so that the diameter of the solid part in the radiological assessment and that of the invasive part in the histological assessment often do not match.

Several studies have validated the new staging system with many cohorts, but they include all histological types together.^4^, ^5^, ^6^, ^7^, ^8^ Wang et al compared the 7th and 8th staging systems in pulmonary adenocarcinoma for clinical stages 0 through IA.^9^ They concluded that the 8th edition predicts postoperative prognosis more precisely than the 7th edition in clinical stage 0‐IA lung adenocarcinoma. Neppl et al compared the 7th and 8th staging systems for primary resected squamous cell carcinomas of the lung and found no significant differences regarding prognostication.^10^ A validation study of the 8th edition TNM classification for resected pulmonary adenocarcinoma of all stages has rarely been reported.

This study aimed to analyze the difference between radiological and pathological tumor sizes, their clinicopathological significance, and the problems in T factor determination in the UICC TNM 7th and 8th classification systems for surgically resected pulmonary adenocarcinoma cases and to evaluate the validity of the 8th edition in a local center.

## 2 PATIENTS AND METHODS

### 2.1 Patient selection

In total, 699 cases of primary lung cancer were resected in our hospital between January 1, 2008, and December 31, 2012. Of these, 478 were adenocarcinoma. After excluding recurrent cases, multiple tumors, cases involving neoadjuvant therapy, metastatic diseases, and other systemic advanced cancers, 429 cases of lung adenocarcinoma with pStage I to III were analyzed. Clinical information, including patient age, sex, smoking history, level of serum CEA before surgery, operative methods, and follow‐up data, were retrieved from medical charts. There were two cases of which cStage was considered to be IIIB. In both cases, clinical T‐descriptor was cT2, and clinical N‐descriptor was not completely sure whether it is N2 or N3. Chemotherapy may usually be prioritized, but surgery was performed at the patient's request. The study was conducted in accordance with the Declaration of Helsinki and approved by our Institutional Research Ethics Committee (No. 1064).

### 2.2 Radiological evaluation

The radiological tumor diameters were measured by an experienced chest radiologist (T. K.) using high‐resolution computed tomography (CT) producing three multi‐detector CT (MDCT) units (GE Healthcare Japan, Siemens Healthineers Japan, Canon Medical Systems Japan). The high‐resolution CT images were reconstructed from the original data using a high spatial resolution algorithm, 1‐mm‐thick slice, and field of view (FOV) focus on the pulmonary nodule. Both the longest‐axis of the tumor and the solid component were measured in each case with a fixed lung window setting (level −600 HU; width 1500 HU), and each longest‐axis served as the reference for the clinical T‐staging of the UICC 7th and 8th editions.

### 2.3 Pathological evaluation

The surgically resected lung nodules were fixed with formalin, and the three‐dimensional sizes of each nodule were measured grossly using a ruler. The longest diameter was recorded as the tumor diameter and served as the reference for pathologic T‐staging of the UICC 7th edition. Histologically, the initial gross measurement was re‐evaluated if significant discrepancies in the longest tumor diameter were observed. The microscopic maximum diameters of invasive lesions were recorded and served as the references for pathologic T‐staging of the UICC 8th edition. If the maximum invasive diameter did not fit into a single block, then the longest invasive diameter was calculated with reference to the gross photograph with a cutting‐out diagram. In the case of invasive mucinous adenocarcinoma, the maximum tumor diameter was also used as the invasive size. We classified the tumors according to the current WHO classification and recorded each growth pattern (lepidic, acinar, papillary, or solid). A tumor with a predominant lepidic growth pattern was graded as grade 1, acinar or papillary pattern was grade 2, and micropapillary or solid pattern was grade 3. Presence or absence of lymphovascular invasion (LVI), and pleural invasion were reviewed and recorded.

### 2.4 Follow‐up information

Follow‐up information was retrieved from the cancer registry of the hospital. The follow‐up period ranged from 35 to 3883 days (mean 2094 days).

### 2.5 Statistical analysis

The Pearson's correlation coefficient was calculated to elucidate the correlation between radiological and pathological sizes. Agreement between clinical and pathological classifications was assessed using weighted k coefficients based on Fleiss‐Cohen weights. Overall survival (OS) rate was determined based on the Kaplan–Meier method in different subgroups and compared using the log‐rank test. Akaike's information criteria (AIC) were used as a discriminative measure for a univariable Cox model for clinical and pathological TNM classifications. Multivariate Cox regression analysis was performed to explore independent prognostic factors. Statistical significance was considered if a two‐sided *p* value <.05 was achieved.

Statistical analyses were performed using StatFlex version 6.0 (Artech, Osaka) for Fisher's exact test, Bland–Altman plot, and Kaplan–Meier method, log‐rank test, and multivariate Cox regression analysis and using BellCurve for Excel (SSRI Co., Ltd., Tokyo) for Fleiss‐Cohen weights.

## 3 RESULTS

### 3.1 Correlation between radiological and pathological sizes and T factors

**FIGURE 1:**
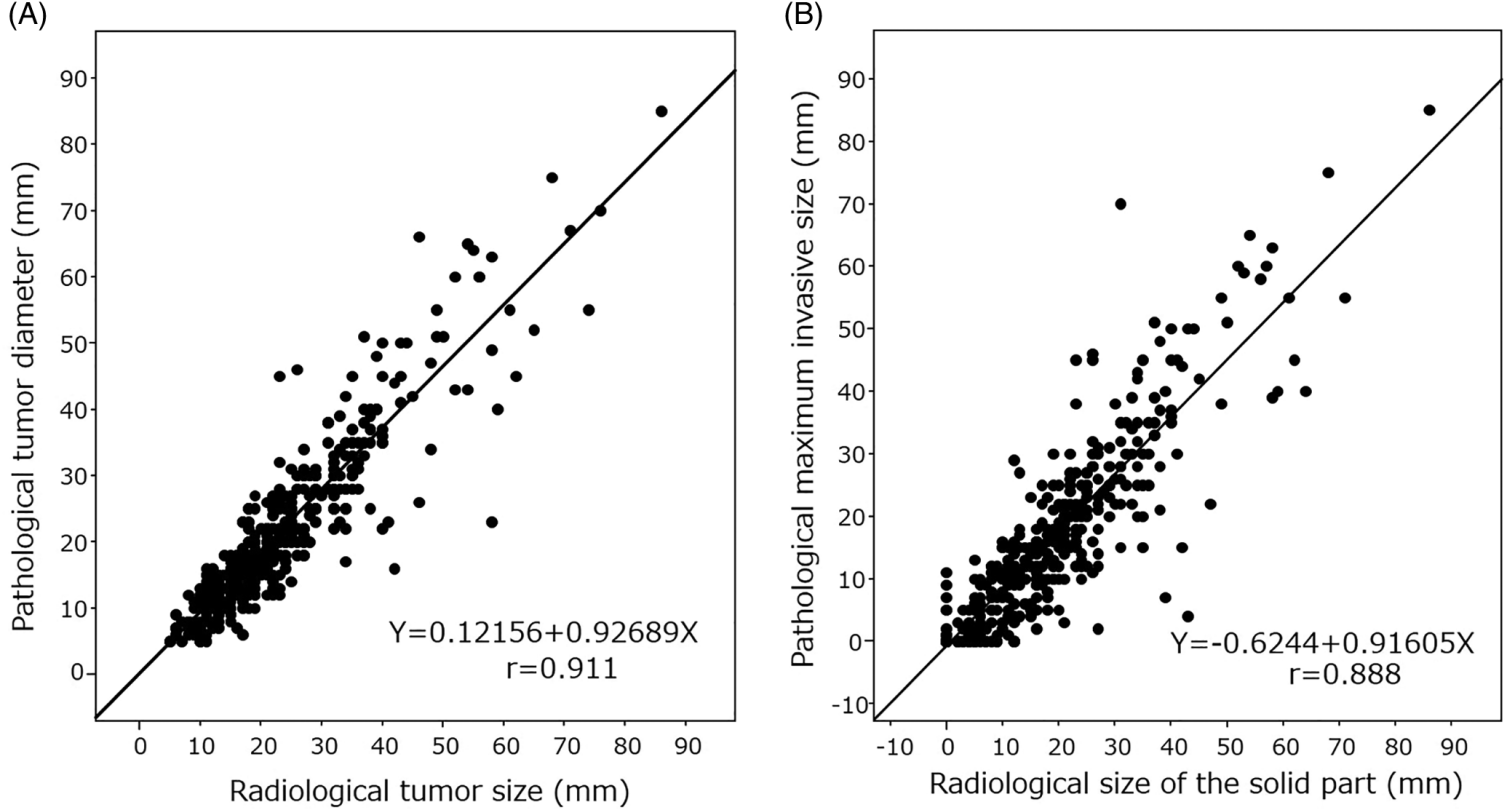
Agreement of computed tomography (CT) and pathology measurement. Scatter plots are of the observed CT measurement (X axis) and pathology measurement (Y axis). Although the correlation coefficient between maximum tumor sizes was slightly higher than that obtained between radiological solid‐parts and pathological invasive diameters (A, *r* = 0.911, *p* < .0001; B, *r* = 0.888, *p* < .0001), the difference was not significant (*p* > .05)

**FIGURE 2:**
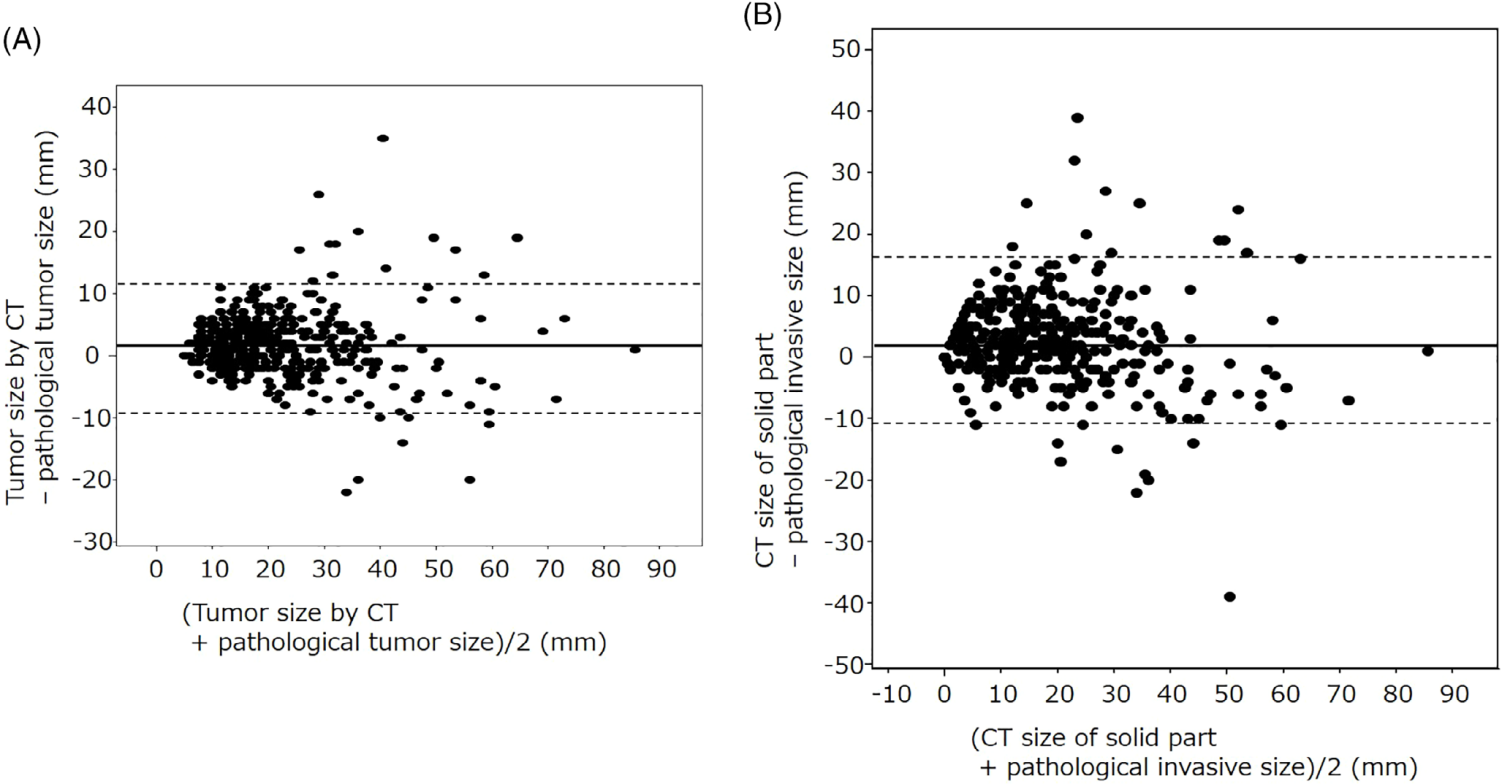
Bland–Altman plot. The mean difference between the two measurements is shown with solid lines at 1.57 mm (A) and 2.10 mm (B); the lower and upper 95% limits of agreement are the dashed lines at −9.25 and 12.39 mm (A) and −11.78 and 15.97 mm (B). The difference between these diameters did not show any significant fixed bias or proportional tendency as there was no apparent pattern on the plot

The mean difference between radiological and pathological maximum diameters was 1.6 mm (SD = 5.4), and the average absolute difference was 3.9 mm (SD = 4.1). The mean difference between radiological solid and pathological invasive diameters was 2.1 mm (SD = 6.9), and the average absolute difference was 4.7 mm (SD = 5.5). The mean difference between the solid‐part and invasive diameters was slightly larger than that between the maximum ones, but the difference was not significant. The correlation coefficient between radiological and pathological maximum diameters was *r* = 0.911 and that between solid‐part and invasive diameters was *r* = 0.888 (Figure 1). Both coefficients showed good correlations. Bland–Altman plots of these differences are shown in Figure 2. The difference between these diameters did not show any significant fixed bias or proportional tendency.

**FIGURE 3:**
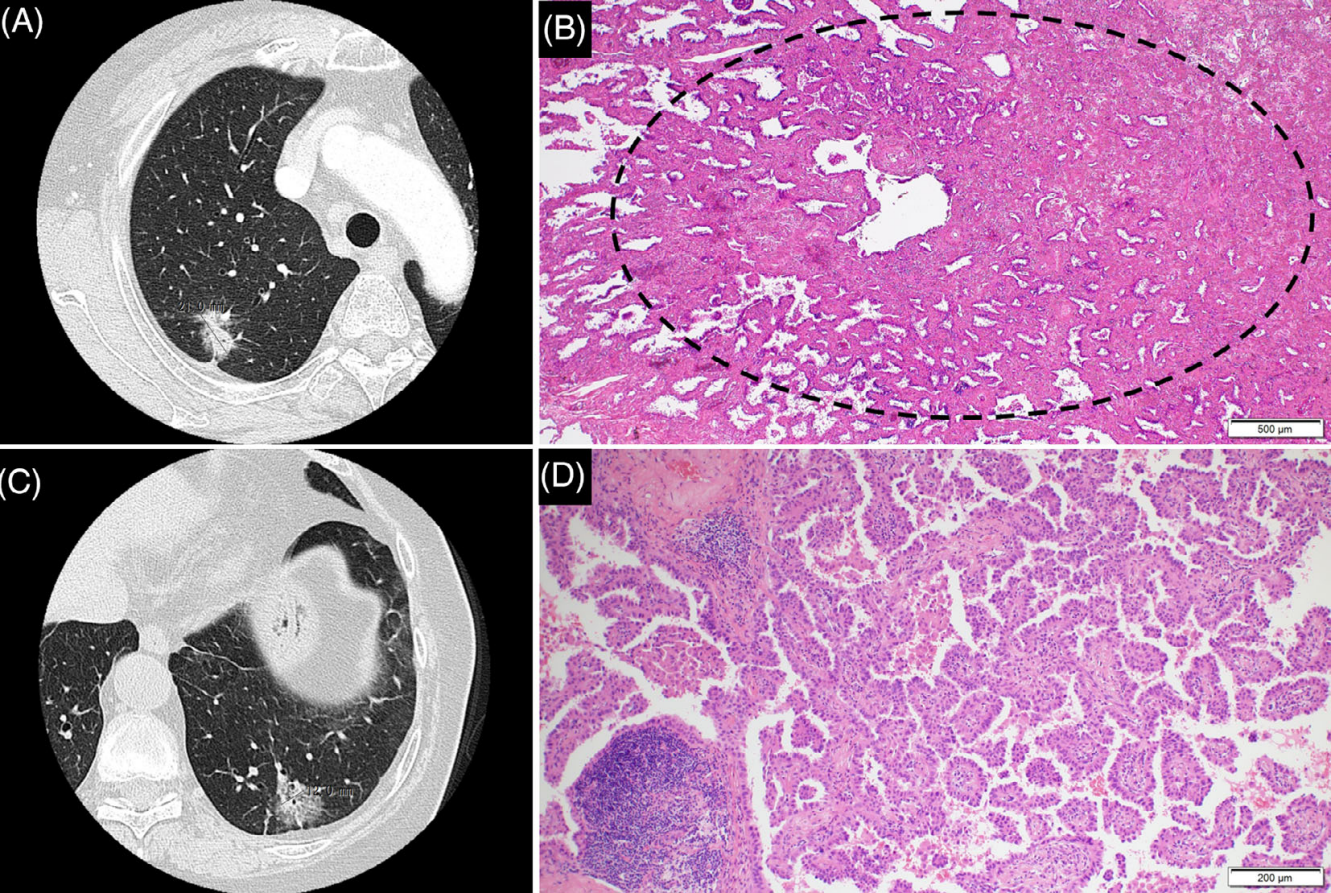
Representative correlation between computed tomography (CT) and histology images. (A). High‐resolution chest CT shows a part‐solid nodule with a solid‐part diameter of 21 mm. (B). Corresponding histology of (A) shows a minimally invasive adenocarcinoma with fibrous scar and an invasive diameter of 3 mm (hemotoxylin and eosin, original magnification ×40). (C). High‐resolution CT shows a part‐solid nodule with a solid‐part diameter of 12 mm. (D). Corresponding histology of (C) shows a papillary predominant adenocarcinoma with an invasive diameter of 29 mm (hemotoxylin and eosin, original magnification ×100)

The main causes for the differences in radiological and pathological tumor diameters were the presence of fibrosis or scarring in the tumor; complications of organizing pneumonia, emphysema, or interstitial pneumonia within or neighboring the tumor; and tumors with a papillary growth pattern (Figure 3). In particular, the presence of fibrosis within or neighboring the tumor tends to make the radiological solid diameter larger than the invasive diameter, and tumors with a papillary growth pattern tend to have an invasive diameter larger than the radiologically‐assessed solid diameter.

The correspondence table between clinical T and pathological T descriptors, and clinical and pathological stages in the 7th and 8th classification systems are shown in Tables S1 and S2. The concordance rates between cT and pT were the highest in T1a in the 7th edition and in cTis in the 8th edition. The rates were the lowest in T1b and T2b in the 7th edition, and in T1mi, T1a, T1c, and T2b in the 8th edition (Figure S1). The weighted kappa coefficients in the 8th edition were superior to those in the 7th edition in both the T and Stage classifications (T7th:T8th = 0.77:0.83, Stage 7th:Stage 8th = 0.67:0.82).

### 3.2 Univariate analysis of each clinicopathological characteristic and overall survival

On univariate analyses, histological grade (G1 vs. G2/3, log‐rank test, *χ* ^2^ = 33.04, *p* < .0001; G1/2 vs. G3, *χ* ^2^ = 20.82, *p* < .0001), age (65 yeras or under 65 years vs. over 65 years, *χ* ^2^ = 11.60, *p* = .00007), sex (female vs. male, *χ* ^2^ = 15.90, *p* = .0001), smoking history (none vs. present, *χ* ^2^ = 13.49, *p* = .0002), preoperative serum CEA level (5.0 ng/ml or less vs. greater than 5.0 ng/ml, *χ* ^2^ = 31.03, *p* < .0001), LVI (absent vs. present, *χ* ^2^ = 50.26, *p* < .0001) were all significant for OS (Figure S2).

**FIGURE 4:**
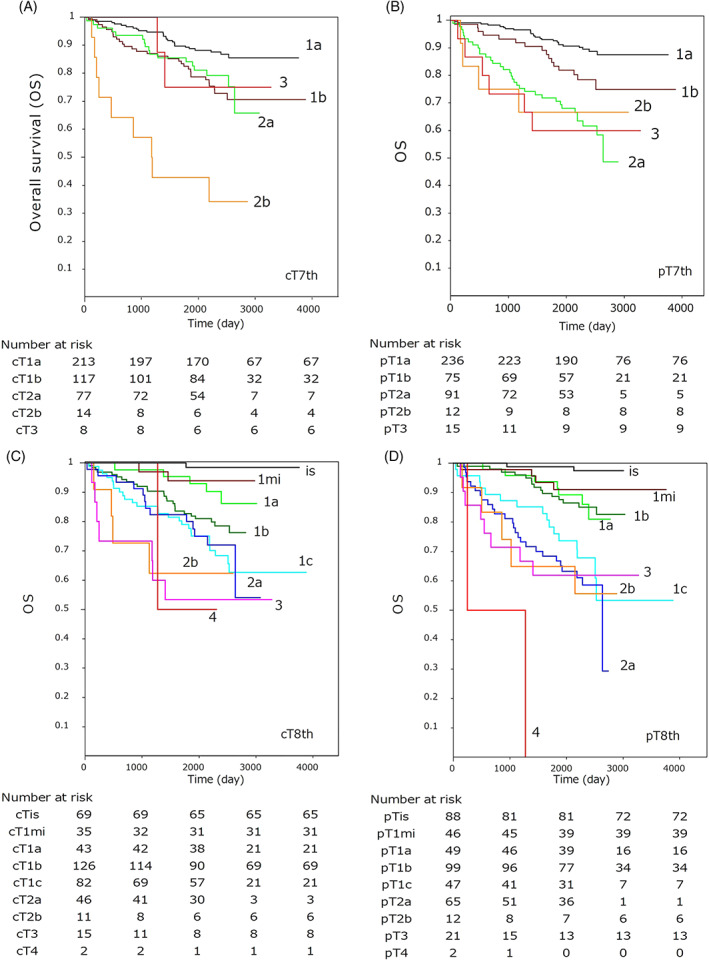
Kaplan–Meier curves showing the overall survival by each clinical and pathological T‐descriptor according to the 7th and 8th editions of the UICC‐TNM classification

**FIGURE 5:**
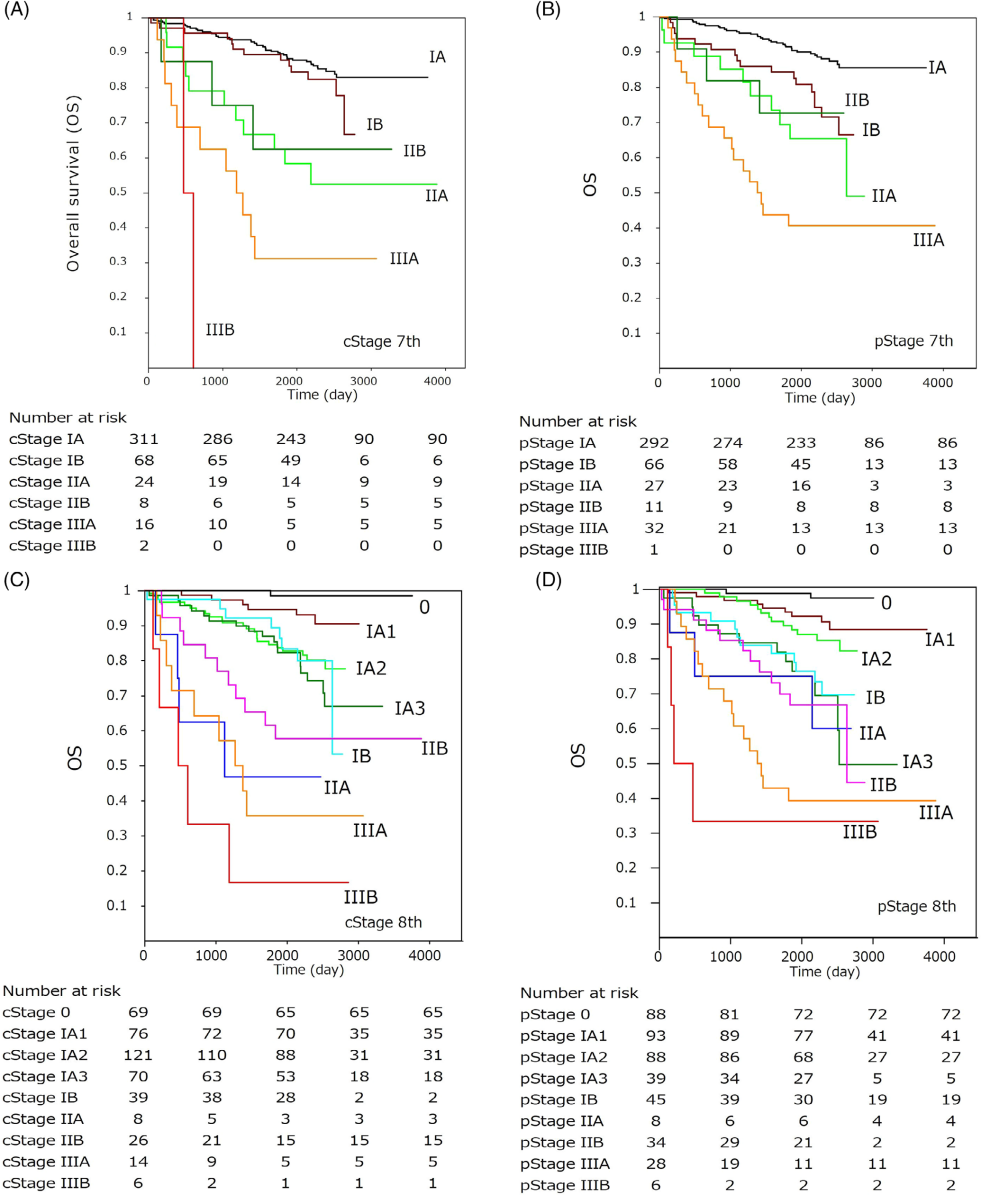
Kaplan–Meier curves showing the overall survival by each clinical and pathological stage according to the 7th and 8th editions of the UICC‐TNM classification

Kaplan–Meier curves for OS by clinical and pathological T descriptors and stages according to the UICC 7th and 8th editions are shown in Figures 4 and 5, and the results of the univariate Cox model for each classification are summarized in Table 1. The AIC levels were lower for the pathological TNM than the clinical TNM, and lower in the 8th edition than in the 7th edition. This result indicates that TNM classification in the 8th edition provided a better model fit than that in the 7th edition in discriminating OS. In the 8th edition, OS among Tis to T1b or T1c to T3 and among Stage 0 to IA2 or Stage IA3 to IIB were not significant both clinically and pathologically. This implies that a solid or invasive tumor size of 2 cm is the critical value for the outcome of pulmonary adenocarcinomas.

**TABLE 1:** Cox proportional hazards model for clinical and pathological TNM classifications per 7th and 8th editions

cStage 7th (AIC 807.71)		95% CI	95% CI		pStage 7th (AIC 805.10)		95% CI	95% CI	
Stage (n)	HR	Lower limit	Upper limit	*p* Value	Stage (n)	HR	Lower limit	Upper limit	*p* Value
IA (261)	Reference				IA (248)	Reference			
IB (64)	1.49	0.81	2.73	0.1992	IB (62)	2.07	1.14	3.74	0.0163*
IIA (21)	3.3	1.6	6.8	0.0012**	IIA (23)	3.31	1.59	6.9	0.0014**
IIB (6)	4.5	1.39	14.58	0.012*	IIB (11)	2.2	0.68	7.15	0.1907
IIIA (8)	10.33	4.35	24.54	<0.001**	IIIA (16)	7.58	3.74	15.36	<0.001**

									
cStage 8th (AIC 785.81)		95% CI	95% CI		pStage 8th (AIC 778.88)		95% CI	95% CI	
Stage (n)	HR	Lower limit	Upper limit	*p* Value	Stage (n)	HR	Lower limit	Upper limit	*p* Value
0 (45)	Reference				0 (48)	Reference			
IA1 (58)	4.15	0.48	35.51	0.194	IA1 (88)	2.46	0.52	11.58	0.2553
IA2 (115)	10.27	1.38	76.22	0.0227*	IA2 (87)	4.06	0.92	18	0.0651
IA3 (69)	14.18	1.89	106.25	0.0099**	IA3 (39)	10.29	2.32	45.68	0.0022**
IB (36)	10.4	1.28	84.6	0.0285*	IB (45)	9.22	2.08	40.91	0.0035**
IIA (6)	73.53	8.18	660.73	<0.001**	IIA (7)	8.15	1.15	57.85	0.036*
IIB (22)	30.23	3.87	236.35	0.0012**	IIB (30)	11.84	2.62	53.47	0.0013**
IIIA (7)	45.02	5.02	403.8	<0.001**	IIIA (13)	26.5	5.61	125.12	<0.001**
IIIB (2)	847.71	68.39	10 506.96	<0.001**	IIIB (3)	507.59	70.8	3638.88	<0.001**

Abbreviations: AIC, Akaike information criterion; CI, confidence interval; HR, hazard ratio.

### 3.3 Multivariate analysis of each clinicopathological characteristic and overall survival

Multivariate analyses of clinicopathological factors using the Cox proportional hazard model were performed using age, sex, smoking history, serum CEA, histological grade, lymphovascular invasion, and cT7th (or pT7th, cT8th, pT8th, cStage7th, pStage7th, cStage8th, and pStage8th). The results revealed that age, LVI, pT8th, and stages in any classification were important determinants of OS, and pStage 8th was the most important, followed by histological grade, CEA level, and sex (Table 2).

**TABLE 2:** Results of multivariate analyses of association clinicopathological factors and overall survival

Variables		HR (95% CI)	*p* Value	Variables		HR (95% CI)	*p* Value
Age	≦65 vs. 65<	2.028 (1.240–3.316)	0.005**	Age	≦65 vs. 65<	2.444 (1.025–4.240)	0.000**
Sex	F vs. M	1.740 (0.901–3.362)	0.099	Sex	F vs. M	1.638 (0.859–3.121)	0.134
Smoking Hx	(−) vs. (+)	1.162 (0.599–2.253)	0.658	Smoking Hx	(−) vs. (+)	1.247 (0.651–2.388)	0.507
Serum CEA (ng/ml)	≦5.0 vs. 5.0<	1.488 (0.937–2.363)	0.092	Serum CEA (ng/mL)	≦5.0 vs. 5.0<	1.610 (1.015–2.554)	0.043*
Histological grade	G1 vs. G2/3	1.745 (0.826–3.685)	0.144	Histological grade	G1 vs. G2/3	2.085 (1.025–4.240)	0.043*
LVI	(−) vs. (+)	2.133 (1.237–3.677)	0.006**	LVI	(−) vs (+)	2.097 (1.219–3.609)	0.008**
pT8th	is‐1b vs. 1c‐4	1.941 (1.115–3.380)	0.019*	pStage 8th	0‐IIB vs. IIIA‐IIIB	3.230 (1.881–5.548)	<0.000***
AIC = 918.93				AIC = 909.15			

Abbreviations: AIC, Akaike's information criterion; F, female; HR, hazrad ratio; Hx, history; LVI, lymphovascular invasion; M, male.

## 4 DISCUSSION

This study showed that the difference between the radiological and pathological diameters was smaller in the UICC 7th edition than in the 8th edition. However, based on the weighted kappa value of the T descriptors, the 8th edition may be a better classification than the 7th edition. In addition, in the univariate Cox model, the UICC 8th classification had a lower AIC value than the 7th classification, and in multivariate analysis, pT8th was a significant factor in addition to the stage factor. These results support the superiority of the 8th classification over the 7th classification in pulmonary adenocarcinoma. Regarding the T‐descriptor subclassification, no significant prognostic difference was noted between Tis to T1b or T1c to T3 in this study. Chen et al stated that T1mi could be included in Stage 0, given that no prognostic difference between Tis and T1mi was identified after comparing Tis in 412 pulmonary adenocarcinomas, T1mi in 675, and IA1 in 437.^11^ Although discriminating T1mi from Tis would bear a difference in the outcome in the long run, the 5‐year survival rate would not differ. It would be an issue in the future to determine the degree of subclassification in early‐stage lung adenocarcinoma that is appropriate in daily practice.

Several larger studies also concluded that the 8th edition predicts postoperative prognosis more precisely than the 7th edition, although they include all histological types together.^4^, ^5^, ^6^, ^7^, ^8^ To the best of the author's knowledge, only two reports compared the validity of the 7th and 8th editions using only adenocarcinoma cases. Although the cases studied were limited to clinical stage 0‐IA lung adenocarcinoma, Wang et al concluded that the 8th edition predicts postoperative prognosis more precisely than the 7th edition.^9^ Kameda et al studied 1704 cases of stage I‐IIA adenocarcinoma, excluding Tis, T1mi, and invasive mucinous adenocarcinoma cases. They showed better c‐index in the 8th edition than in the 7th edition and concluded that the 8th edition is superior as the prognostic discriminator. We studied a relatively small number of cases, but we included Tis, T1mi, and invasive mucinous adenocarcinoma cases. In our study, the 8th edition also seemed to be a better model for TNM classification.

The radiological and pathological tumor diameters showed high correlation rates, and the correlation coefficients in the 7th and 8th editions were 0.911 and 0.888, respectively. Bland–Altman plots did not show any significant fixed bias or proportional tendency. The mean diameter difference was larger in the 8th edition, and the more the T‐descriptor is subdivided, the lower is the concordance rate between cT and pT. Although weighted kappa was superior in the 8th edition, cT and pT need to be matched as much as possible.

The major reasons for the difference between radiological solid diameter and pathological invasive diameter were the presence of intratumoral or peritumoral fibrosis or organization, and a fair amount of papillary pattern. Although a papillary pattern is regarded as invasion histologically, the pattern retains air spaces in the tumor, which appears in non‐solid pattern radiologically. Therefore, the radiological differentiation of it from a lepidic growth pattern will be extremely difficult. Some reported that the predominantly lobulated configuration in adenocarcinoma contained more papillary and micropapillary growth patterns.^12^, ^13^ Therefore, when lobulated margins are observed in a ground‐glass‐like nodule, the possibility of papillary adenocarcinoma should be considered, and using PET/CT would further help to differentiate between papillary adenocarcinoma and lepidic adenocarcinoma.^14^, ^15^ Under the current radiological conditions, differentiation between fibrous tissue and invasive tumor will be almost impossible; hence, the emergence of a new modality is awaited.

Other possible factors influencing the difference between radiological solid diameters and pathological invasive diameters are as follows: first, as physiological factors, a state at the time of image shooting and physiological tissue shrinkage are raised.^16^ Second, as a radiological factor, a difference in the recognition of the solid part by each radiologist is an important factor. Third, as surgical factors, time to fixation (warm and cold ischemic time), and fixation with staplers (collapsing) may influence the tumor diameter.^17^ Fourth, pathological factors include (a) fixation time, (b) fixation artifact (collapse, elongation by pressing), (c) failure to measure the actual maximum diameter, and (d) differences in the evaluation of the invasive part by each pathologist are raised. To achieve standardization, radiologists should further make efforts to standardize the method of evaluating the solid part in images. Surgeons have to make appropriate fixation as soon as possible after surgery. Pathologists should perform cutting the tissue properly, standardize the criteria to evaluate invasion, and universalize the method for measuring the invasive size. Taking a gross photograph of the tumor cross‐section to allow for correlation with microscopic findings will help to measure the maximum invasive diameter.

Radiologically, a high inter‐observer agreement was seen for solid nodules, and the majority of disagreements were related to subsolid nodules, including either the presence or absence of a solid component, and the size of the solid component.^18^ Inter‐observer agreement on nodule classification into pure ground‐glass nodules and part‐solid nodules was moderate to excellent (mean kappa 0.51–0.87).^18^, ^19^, ^20^ The results indicate that the evaluation of a potential solid component within a nodule containing ground glass components is prone to substantial interobserver variability. This variability is likely caused by the subjective nature of the task in the absence of absolute measurements. The current consensus is that such nodules are best evaluated subjectively using a lung window setting and a high‐spatial‐frequency (sharp) filter to judge the presence and extent of solid components.^21^, ^22^ Some studies have shown that using the mediastinal window setting to assess the solid portion of lung cancer could improve the inter‐observer agreement in classifying subsolid lung nodules.^19^, ^20^ Meanwhile, some authors found that measuring the solid component of nodules with lung window better correlated with histological evidence of tumor invasion than other window settings.^22^, ^23^, ^24^ Recent studies have shown that a semiautomatic measurement could improve the inter‐observer agreement for subsolid nodules,^25^ and quantitative analysis of CT attenuation value may help distinguish invasive adenocarcinoma from noninvasive ones.^26^ Regular use of these methods might be time consuming, but in selected cases, it would contribute to a decrease in inter‐observer variability.

Pathologically, Thunnissen et al assessed the reproducibility of invasion and non‐invasion of lung adenocarcinoma among an international group of pulmonary pathologists and showed that there was moderate reproducibility for typical cases (*κ* = 0.55) and slight reproducibility in difficult cases (*κ* = 0.08).^27^ Noguchi et al reported that 27 Japanese general pathologists evaluated noninvasive and invasive adenocarcinomas using 32 small adenocarcinomas. Their average inter‐observer concordance rate changed from 80.3 to 85.3% after taking the educational program, while the inter‐observer agreement of six pulmonary pathologists was 89%.^28^ More reliable diagnostic methods and the development of international enlightenment and educational activities for pathologists will be necessary to reach an agreement regarding invasion and non‐invasion.^29^

On the other hand, Matsuguma et al reported that regardless of the solid area diameter, no patient with a greater proportion of ground glass opacity (GGO; >50%) experienced recurrence, and that the proportion of GGO was more significantly associated with disease‐free survival (DFS) than solid diameter.^30^ The study by Kadota et al pathologically backed up these results in that all cases of AIS, MIA, and stage I lepidic adenocarcinomas that had a lepidic component of more than 50% did not recur for 5 years.^31^ Therefore, in lepidic‐predominant small adenocarcinomas, the presence or absence of small invasive foci would not be critical from a clinical perspective.

Many researchers have reported that in stage I adenocarcinoma, disease‐free survival or lymph node metastasis was significantly better in patients with part solid nodules than in patients with solid nodules without GGO.^32^, ^33^, ^34^ Although a few researchers claimed that there were no significant prognostic differences between them,^25^ nodule classification such as non‐solid, part‐solid, or solid nodule might have to be incorporated in the T‐descriptor for small adenocarcinomas in the future. Recently, several radiological nomograms integrating various factors such as size, shape, regularity, and CT attenuation have been advocated to predict the invasiveness of subsolid nodule pulmonary adenocarcinomas.^35^, ^36^, ^37^ Taking proper advantage of this information will be another challenge.

The present study has some limitations. This was a retrospective study performed at a single institution and had a relatively small sample size. We did not have information on the gene mutational status in most cases.

## 5 CONCLUSION

From this study, pulmonary adenocarcinoma classification by the 8th edition appears to be superior to the 7th edition. However, in adenocarcinoma, unlike small cell carcinoma and squamous cell carcinoma, in which the tumor diameter is equal to the invasive diameter, there are differences between the solid diameter and the invasive diameter due to various factors, and the possibility of a difference between cT and pT increases. In addition, the degree of significance of the subclassification in T‐descriptors may depend on the histological type. As for adenocarcinomas showing subsolid nodules, more reliable diagnostic methods and continuous efforts are needed to increase the universality of the measurement of the radiological maximum solid diameter and the pathological maximum invasive diameter. At the moment, pathologists should always consider performing the radiologic‐pathologic correlation for determining the invasive size.

## CONFLICT OF INTEREST

The authors declare there is no conflict of interest.

## AUTHOR CONTRIBUTIONS

All authors had full access to the data in the study and take responsibility for the integrity of the data and the accuracy of the data analysis. Each author's contribution is followed as—*Conceptualization*, H. M.; *Methodology*, H. M.; *Software*, H. M.; *Validation*, K. K., H. K., Y. T., T. K.; *Investigation*, H. M., T. K.: *Formal analysis*, K. K., T. K.; *Resources*, N. T., H. F., Y. T.; *Writing ‐ Original Draft*, H. M.; *Writing ‐ Review & Editing*, H. M., T. K.; *Supervision*, H. K.

## ETHICAL STATEMENT

The study was conducted in accordance with the Declaration of Helsinki and approved for the retrospective study by our Institutional Research Ethics Committee (No. 1064). Patients were not required to give anew informed consent to the study because the analysis used anonymous clinical data that were obtained after each patient agreed to treatment by written consent. And also, we applied opt‐out method to obtain consent on this study through the website.

## Supporting information


**Figure S1** Complete match rate between cT and pT‐descriptors. The weighted kappa coefficients were 0.77 (95% CI: 0.72–0.83) in the 7th edition and 0.82 (95% CI: 0.79–0.86) in the 8th edition.

**Figure S2** Kaplan–Meier overall survival curves for each factor. A. Age, B. tumor grade, C. sex, D. smoking history, E. preoperative serum CEA level, F. lymphovascular invasion.

**Table S1** The correspondence table between clinical T‐ and pathological T‐descriptors in the seventh edition of UICC

## Data Availability

The datasets used and/or analyzed during the current study are available from the corresponding author on reasonable request.
