# Supplementary figures and images for: Verification of the eighth edition of the UICC‐TNM classification on surgically resected lung adenocarcinoma: Comparison with previous classification in a local center
Source: Cancer Rep (Hoboken). 2021 Jun 24;5(1):e1422. doi: 10.1002/cnr2.1422 (PMC8789611; doi:10.1002/cnr2.1422)

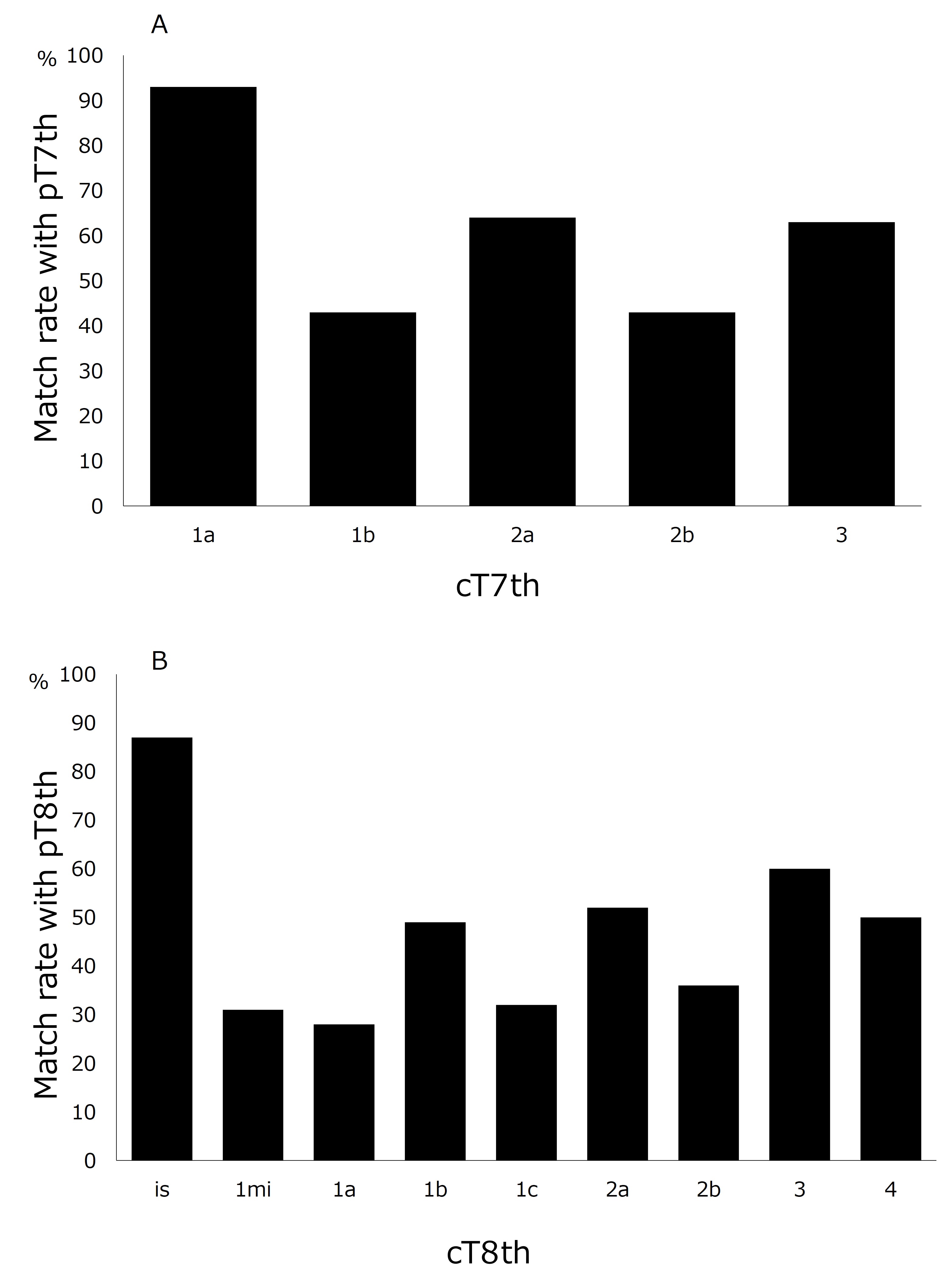

Supplement: Supplementary file 1 — Figure S1 Complete match rate between cT and pT‐descriptors. The weighted kappa coefficients were 0.77 (95% CI: 0.72–0.83) in the 7th edition and 0.82 (95% CI: 0.79–0.86) in the 8th edition. [file CNR2-5-e1422-s002.jpg]

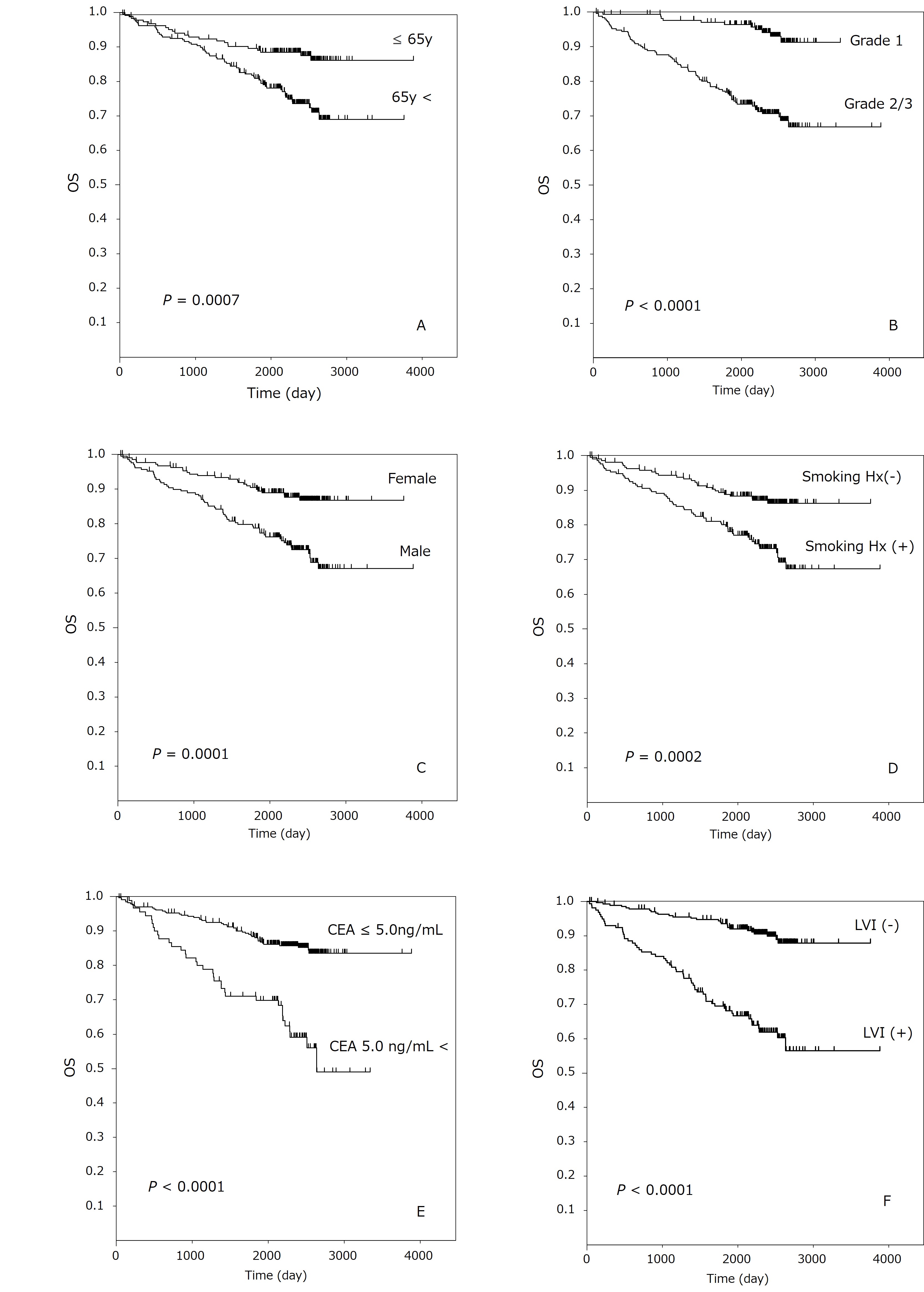

Supplement: Supplementary file 2 — Figure S2 Kaplan–Meier overall survival curves for each factor. A. Age, B. tumor grade, C. sex, D. smoking history, E. preoperative serum CEA level, F. lymphovascular invasion. [file CNR2-5-e1422-s003.jpg]
